# Health inequalities and the influence of economic development, renewable energy, financial growth, and resource use in emerging economies

**DOI:** 10.3389/fpubh.2025.1658192

**Published:** 2025-09-10

**Authors:** Xin Jin, Farzana Akram

**Affiliations:** ^1^Teaching and Research Department of Social and Ecological Civilization, Party School Gansu Committee of C.P.C, Lanzhou, China; ^2^Department of Chemistry, University of Lahore, Lahore, Pakistan

**Keywords:** renewable energy consumption, natural resource rents, adult literacy rate, access to improved water sources, public health, health disparities

## Abstract

This study investigates the heterogeneous effects of economic level, renewable energy consumption, financial development, natural resource rents, adult literacy, and access to improved water sources on life expectancy at birth in Emerging Seven (E7) economies. Employing the Method of Moments Quantile Regression (MMQR), the analysis examines these relationships across the 25th, 50th, 75th, and 90th percentiles to capture distributional variations in health outcomes. The findings indicate that economic level and renewable energy consumption have positive but diminishing effects on life expectancy at higher percentiles, while financial development exerts a consistently strong and increasing influence across all levels. Natural resource rents demonstrate a persistent negative association, underscoring the risks of resource dependence. In contrast, adult literacy and access to improved water sources emerge as robust determinants of health, with their positive effects strengthening at higher percentiles. These results suggest that economic and environmental progress alone are insufficient without parallel investments in human capital and basic infrastructure. The study recommends an integrated policy framework that strengthens financial systems, promotes sustainable energy use, enhances education and water access, and ensures responsible resource governance to advance long-term health outcomes and inclusive development across E7 countries.

## 1 Introduction

Improving life expectancy remains a central goal for policymakers seeking to promote sustainable development and inclusive growth. In many Emerging Seven (E7) economies, rapid economic expansion has been accompanied by persistent health disparities, underscoring the need to understand the drivers of population health. While economic growth and renewable energy adoption can contribute to better health outcomes, these gains are often unevenly distributed and insufficient on their own (1, 2). Factors such as financial development inflows, natural resource rents, adult literacy rates, and access to improved water sources also play critical roles in shaping life expectancy. Examining these diverse influences is essential to design effective policies that not only advance economic and environmental goals but also improve public health and reduce inequality.

The dynamic interplay between economic level (ECL), renewable energy consumption (REC), financial development (FDI), natural resource rents (NRR), adult literacy rate (ALR), and access to improved water sources (AIWS) plays a crucial role in shaping human well-being and sustainable development in E7 countries (3, 4). The E7 group comprises China, India, Indonesia, Brazil, Russia, Mexico, and Turkey—the seven largest emerging economies in terms of size and growth potential. Life Expectancy at Birth (LEX) serves as a vital measure of public health, providing insight into general living standards and the performance of healthcare systems (5). It is an essential indicator of quality of life, as it closely relates to the availability of healthcare services, adequate nutrition, and a clean-living environment (6). LEX is not only a key metric for evaluating a country’s health outcomes but also acts as a mirror of its economic advancement and social development (7). The Sustainable Development Goals (SDGs) highlight the significance of enhancing LEX, especially through SDG 3 (Good Health and Well-Being) and SDG 6 (Clean Water and Sanitation). Economic level (ECL) is a key driver in this process, as it generates the financial means to invest in healthcare systems, social support services, and overall living conditions (8). A higher ECL allows governments to direct more resources toward health systems and public services, thereby boosting life expectancy by improving access to healthcare and raising overall living standards (9). However, the link between ECL and LEX is complex. Although economic level can enhance life expectancy by enabling greater investments in healthcare and education, unregulated or uneven growth may widen social inequalities, negatively impacting the health and well-being of marginalized groups.

Access to renewable energy consumption (REC) also plays a significant role in shaping LEX. Clean energy sources like solar, wind, and hydroelectric power support environmental sustainability by lowering air pollution and helping to combat climate change (10). Such environmental improvements can directly benefit public health by reducing the prevalence of pollution-related diseases, thereby contributing to longer life expectancy (11). Furthermore, clean energy sources can provide reliable power to remote or underserved areas, enhancing healthcare delivery and improving general living conditions. SDG 7 (Affordable and Clean Energy) underscores the importance of transitioning to clean energy, which can positively influence LEX by fostering healthier environments.

Financial development (FDI) plays a vital role in enhancing LEX by facilitating access to capital needed for investments in healthcare, education, and essential infrastructure (12). Effective financial systems improve access to credit, strengthen banking services, and promote capital market growth, all of which are essential for funding public health programs and medical research. Financial development is also associated with improved health outcomes, as greater financial stability allows governments and individuals to invest in healthier lifestyles, enhanced healthcare, and better sanitation. Research has demonstrated that financial development can help reduce health disparities by increasing access to health services and lowering economic barriers to care (13). When managed sustainably, natural resource rents (NRR) can positively influence life expectancy by supplying economic resources that support development while preserving environmental health (14). However, the overexploitation of natural resources can cause environmental degradation, which adversely impacts public health and consequently reduces life expectancy. Exploring the connections between environmental degradation and economic level reveals critical pathways through which unsustainable resource use undermines health outcomes and long-term development (15). Unsustainable use of resources leads to pollution, deforestation, and climate change, which can cause respiratory illnesses, water shortages, and various other health problems, ultimately lowering life expectancy at birth (LEX) (16). Effective management of NRR is crucial to promoting long-term health and sustainability, instead of worsening environmental and health problems.

In addition to these factors, this study incorporates Adult Literacy Rate (ALR) and Access to Improved Water Sources (AIWS) as critical determinants of life expectancy. Education, captured through ALR, improves health outcomes by raising awareness of hygiene, nutrition, and disease prevention, while supporting economic opportunities and social equity. Access to improved water sources is a fundamental component of public health infrastructure that reduces exposure to waterborne diseases and improves overall living standards. Including ALR and AIWS in the analysis ensures a more comprehensive understanding of the human capital and infrastructure dimensions that shape health outcomes, acknowledging their importance for sustainable development in emerging economies.

This study seeks to analyze the impact of ECL, REC, FDI, NRR, ALR, and AIWS on life expectancy at birth (LEX) in E7 countries. Gaining insight into these connections can help policymakers design strategies that support sustainable economic development and enhance public health. Considering the significant demographic and economic potential of E7 nations, examining how economic level, renewable energy use, financial development, natural resource management, education, and basic infrastructure affect LEX is essential for achieving both social and economic advancement. This study contributes to literature in several important ways. First, it simultaneously examines economic level, renewable energy consumption, financial development, and natural resource rents as determinants of life expectancy, offering a multidimensional framework rarely applied to emerging economies. Second, it focuses specifically on the E7 countries large and fast-growing economies that are understudied compared to OECD or global samples thereby filling an important geographical gap. Third, by applying the Method of Moments Quantile Regression (MMQR), the analysis captures distributional heterogeneity in life expectancy, moving beyond mean-based approaches commonly used in prior work. Finally, the study links empirical findings with concrete policy insights, emphasizing the intersection of economic, environmental, and health dimensions. Together, these contributions highlight the novelty of this research relative to existing studies.

ECL, REC, FDI, NRR, ALR, and AIWS all play a role in shaping economic, technological, and social progress, each contributing to better living standards and health outcomes. As emerging economies, E7 countries have experienced notable changes in their financial systems, energy sectors, education levels, and infrastructure access. These shifts have directly affected public health and life expectancy, underscoring the need to align health, economic, and environmental policies. This study will examine how these factors interact to impact LEX and offer policy recommendations to enhance life expectancy and promote sustainable development in E7 nations.

This study focuses on the E7 countries (China, India, Indonesia, Brazil, Russia, Mexico, and Turkey) because they represent the world’s seven largest emerging economies with rapidly growing populations, diverse natural resource endowments, and significant contributions to global economic output. The E7 economies are projected to surpass the G7 in aggregate GDP in the coming decades, making their development trajectories critical for global sustainability and public health (17). These countries also exhibit substantial heterogeneity in economic level, renewable energy adoption, financial development, natural resource management, education levels, and infrastructure quality, providing a valuable context for examining how these factors jointly influence life expectancy at birth. By concentrating on E7, this study offers targeted insights that can inform development policy and public health strategies in major emerging markets where improvements in life expectancy have significant global implications.

Unlike much of the existing literature, which predominantly estimates mean effects, this study employs the Method of Moments Quantile Regression (MMQR) to capture the heterogeneous impacts of economic, financial, environmental, and social factors on life expectancy across its entire distribution (18). This approach is crucial because mean-based methods may obscure differences across population groups, thereby underestimating or misrepresenting the true nature of health inequalities. By focusing on distributional variations, the study contributes a more precise understanding of how development-related determinants shape health outcomes in the E7 economies, thereby offering stronger evidence for targeted and inclusive policy interventions.

This study makes a novel contribution by integrating economic, energy, and financial dimensions with public health outcomes in the context of the Emerging Seven (E7) economies. While previous research has often examined these relationships in isolation or focused primarily on advanced economies, evidence from major emerging countries remains limited. By jointly analyzing these interconnections, the study not only provides timely insights into the determinants of life expectancy but also advances the literature on health equity and environmental health in developing contexts.

The rest of this study is organized as follows: Section 2 surveys the empirical literature on the links between LEX, ECL, REC, FDI, NRR, ALR, and AIWS. Section 3 details the data sources, methodological framework, and estimation techniques. Section 4 presents the findings and offers policy recommendations.

## 2 Literature review

Reaching an adequate level of Life Expectancy at Birth (LEX) to tackle global economic and social challenges is among the most urgent issues today. Sustainable development defined as an economic level that respects the planet’s ecological limits is crucial for long-term advancement. Yet, existing global development trends are predominantly unsustainable, highlighting the need for immediate reforms to better align economic level with environmental and social sustainability (19). A vital aspect of this transformation is improving LEX, as it significantly contributes to greater productivity, innovation, and overall societal welfare.

### 2.1 The impact of ECL on life expectancy

Gross Domestic Product (ECL) is widely regarded as a major engine of economic development, shaping the resources available to enhance life expectancy at birth (LEX). Policies that encourage technological advancement, boost business productivity, and generate employment have a direct effect on public health by improving access to healthcare, sanitation, and education. With higher ECL, governments typically have greater capacity to invest in infrastructure and healthcare systems, ultimately leading to longer life expectancy (20). However, although it is often believed that countries rich in natural resource rents (NRR) and renewable energy consumption (REC) will achieve rapid ECL growth, this expectation does not always hold true (21). It is important to recognize that economic level does not automatically stem from resource abundance; in fact, excessive dependence on natural resource extraction can hinder economic diversification, ultimately limiting its long-term benefits for public health and life expectancy (22).

Empirical research also highlights the positive link between trade openness, life expectancy at birth (LEX), and ECL. A panel analysis of Balkan countries from 2000 to 2019 revealed that trade openness and LEX both had significant positive effects on ECL growth, underscoring their role in enhancing economic performance and promoting public health (23). Likewise, in BRICS countries, financial development (FDI) inflows have been mainly fueled by ECL growth, while factors like political stability and life expectancy at birth (LEX) serve a supporting role in advancing public health by raising living standards (24). LEX and ECL growth reinforce each other in attracting foreign investment, indicating that strengthening health and social infrastructure can boost economic stability while also increasing life expectancy (25).

### 2.2 Financial development and its impact on life expectancy

The relationship between financial development (FDI) and life expectancy at birth (LEX) has been extensively examined, especially in the context of developing countries (26). Financial development is essential for enhancing healthcare access by enabling greater investment in the health sector, expanding educational opportunities, and supporting overall well-being. (27) examined the uneven relationship between FDI, ECL, and LEX in ASEAN countries, showing that although both ECL and FDI have positive impacts on LEX, the long-term effects of FDI are asymmetric. Nwani (28) showed that over the long term, Nigeria’s ECL is strongly affected by LEX, supporting the view that better public health directly fosters sustainable economic level.

Moreover, Ibrahim (29) revealed that although rising income levels in Africa are linked to environmental degradation, advancements in trade, life expectancy at birth (LEX), and renewable energy consumption (REC) improve environmental quality by lowering the ecological footprint. This indicates that LEX serves as a mediating factor in reconciling economic level with environmental sustainability. Xu et al. (17) also suggest that LEX acts as a catalyst in enhancing the positive impact of REC on ECL, highlighting the strong link between renewable energy consumption, public health, and economic level. Recent studies also highlight the role of green finance in promoting energy efficiency and supporting sustainable urban development, underscoring the importance of financial sector development for achieving environmental and health goals (30). Financial development is essential for enhancing healthcare access by enabling greater investment in the health sector, expanding educational opportunities, and supporting overall well-being. This underscores the critical role of financial development in advancing both health outcomes and environmental sustainability.

### 2.3 The role of natural resource rents in life expectancy

In emerging economies rich in natural resource rents, low life expectancy at birth (LEX) is frequently linked to the resource curse, where heavy reliance on resource extraction results in economic stagnation and environmental harm, ultimately lowering life expectancy (31). The United Nations Development Program points out that overexploitation of resources has led to serious environmental issues—such as deforestation, soil erosion, and loss of biodiversity especially in emerging economies like the E7 and N11 countries (32). However, improvements in LEX can help turn the resource curse into an economic benefit by fostering knowledge-driven industries and decreasing dependence on raw material exports (33). Nonetheless, Lyatuu et al. (34) warn that mineral rents may adversely affect LEX development, depending on how heavily a country’s economy relies on them. In this study, natural resources are operationalized as natural resource rents as a percentage of GDP, capturing the economic value derived from extractive industries. While governance quality is widely recognized as a critical factor moderating the resource–health relationship, it is not directly included in our model, and this limitation is acknowledged in our analysis.

### 2.4 Economic growth and health

Previous studies generally confirm a positive association between economic growth and life expectancy, suggesting that higher income levels improve access to healthcare, nutrition, and living standards. However, most of these studies estimate only the mean effect of income on health, leaving unanswered whether the benefits of economic development are equally shared across populations with different health outcomes (35). This raises the question of whether economic growth reduces or reinforces health inequalities an issue this study seeks to address.

### 2.5 Renewable energy and health

A growing body of work links renewable energy consumption with improved health outcomes, primarily through reductions in air pollution and environmental degradation (36). While these findings are consistent, the literature has not fully explored whether the health benefits of renewable energy are uniform across populations. Little is known about whether renewable energy consumption benefits those with already higher life expectancy than those with lower life expectancy, which is a critical dimension of health inequality.

### 2.6 Finance and health

Financial development has been shown to improve health outcomes by increasing access to capital, promoting healthcare investment, and fostering inclusive growth (35). Yet, studies disagree on whether financial flows benefit the entire population equally or disproportionately favor specific groups. The distributional impacts of finance on health remain unclear, which justifies investigating these effects across different life-expectancy levels.

### 2.7 Natural resources and health

Research on natural resources suggests that rents from resource exploitation often undermine health outcomes by fostering economic volatility, weak institutions, and environmental harm. Nonetheless, the evidence is mixed, with some studies indicating positive effects under strong governance (37). These inconsistencies highlight the importance of examining how resource dependence affects health across different segments of the population, rather than only on average.

### 2.8 Social indicators and health

Education and access to clean water are widely recognized as fundamental drivers of health improvement (35). However, existing studies often examine these variables as controls rather than as central determinants. Moreover, their distributional effects across different health levels have not been systematically investigated, despite their critical role in reducing health inequalities.

### 2.9 Impact of this research

Given the varied economic conditions and resource endowments among E7 countries, it is essential to analyze how ECL, REC, FDI, and NRR influence LEX in this group. This study explores these relationships over the period 2000 to 2022, using advanced econometric methods such as stability analysis, matrix correlation, CIPS unit root tests, slope heterogeneity, Westerlund cointegration, and Method of Moments Quantile Regression (MMQR) to ensure robust and reliable results. By examining the key factors affecting LEX, this research offers important insights for policymakers to develop strategies that support economic level while prioritizing public health and human well-being.

Ultimately, a country’s development and prosperity depend on its Life Expectancy at Birth (LEX), which serves as a broad indicator of public health and overall well-being. The economic level is strongly linked to rising LEX, highlighting the importance of understanding how ECL, REC, FDI, and NRR impact health outcomes. This literature review emphasizes the need for a balanced strategy that combines economic level, responsible resource management, and sustainable public health initiatives to achieve lasting national and global progress.

## 3 Theoretical basis

This research explores the complex relationships between Gross Domestic Product (ECL), Renewable Energy Consumption (REC), Financial Development (FDI), Natural Resource Rents (NRR), and Life Expectancy at Birth (LEX) in E7 nations from 2000 to 2022. The study is anchored in key economic, environmental, and development theories to clarify how these variables jointly shape life expectancy outcomes.

The link between economic level (ECL) and Life Expectancy at Birth (LEX) is based on modernization theory, which suggests that economic development leads to better public health by raising incomes, improving access to healthcare, and enhancing overall living conditions. According to Miladinov (38), ECL growth increases government revenues, enabling greater investment in healthcare infrastructure, sanitation, and nutrition, critical factors that influence life expectancy. Furthermore, as economies expand, they typically allocate more resources to social welfare programs, resulting in better healthcare outcomes (39).

However, the connection between ECL and LEX is not straightforward. Although rising income usually enhances life expectancy, the environmental damage and social inequalities that often accompany rapid growth can harm public health. For example, economic expansion fueled by resource extraction or intensive industrialization can cause pollution and negative health effects, as noted by Sun et al. (40). Thus, the impact of ECL on life expectancy depends on the quality and inclusivity of growth, which in turn depends on the management of resources and economic policies.

The relationship between Renewable Energy Consumption (REC) and Life Expectancy at Birth (LEX) can be understood through sustainability and environmental health theory. According to Stern (41), the transition to renewable energy consumption sources—such as solar, wind, and hydroelectric power—has a direct impact on public health by reducing the environmental pollution associated with fossil fuel consumption. Cleaner energy sources improve air quality, reduce respiratory illnesses, and decrease premature deaths related to pollution. Therefore, greater adoption of renewable energy consumption is expected to contribute positively to life expectancy by mitigating environmental risks.

Moreover, renewable energy consumption adoption is essential for long-term sustainable development. The Green Growth Theory suggests that economic development that prioritizes environmental sustainability can lead to improved health outcomes by reducing environmental risks (42). As E7 countries shift toward renewable energy consumption, they reduce their dependence on fossil fuels, mitigating the negative health effects of air pollution and fostering a healthier population.

Financial development (FDI) plays a crucial role in improving life expectancy through its impact on health financing, education, and overall social welfare. The financial development theory argues that an efficient financial system facilitates the allocation of resources for investments in health infrastructure, education, and social services (43). As financial markets grow and become more accessible, countries can mobilize domestic savings and attract foreign capital to invest in public health initiatives, thereby improving life expectancy outcomes. In addition to economic, financial, and environmental determinants, social infrastructure factors such as Adult Literacy Rate (ALR) and Access to Improved Water Sources (AIWS) also play critical roles in shaping life expectancy. Adult literacy enhances individuals’ ability to understand health information, adopt preventive behaviors, and make informed medical decisions. It also improves employability and income, which in turn provide better access to healthcare and nutrition. These mechanisms are consistent with Human Capital Theory, which emphasizes the role of education in improving productivity and health. Likewise, access to improved water sources reduces the spread of waterborne diseases such as cholera, diarrhea, and typhoid, which are major causes of mortality in developing economies. Clean water also supports hygiene, food safety, and nutritional security, thereby strengthening population health over the long term. Together, ALR and AIWS capture the importance of social determinants of health, complementing economic, financial, and energy-related factors in explaining variations in life expectancy across E7 countries.

Additionally, financial development can improve households’ access to credit, allowing them to obtain healthcare services and maintain healthier living environments (44). It has been shown that in countries with advanced financial systems, access to healthcare expands and public health outcomes improve due to greater capacity to finance health services and programs. Consequently, financial development serves as an intermediary between economic level and life expectancy, enabling better health outcomes.

The influence of Natural Resource Rents (NRR) on life expectancy is rooted in the resource curse theory, which argues that excessive reliance on resource extraction can produce adverse economic and social consequences such as environmental damage, greater income inequality, and deteriorating public health (45). It suggests that resource-rich countries may struggle to diversify their economies, resulting in economic instability and underdevelopment. In these situations, revenues from natural resource rents are often not effectively allocated to social sectors such as healthcare, contributing to weaker health outcomes and reduced life expectancy.

Alternatively, a more refined perspective, referred to as the resource blessing theory (46), suggests that, when effectively managed, natural resource rents can drive economic level and development. Nations that wisely steward their resource wealth can channel investments into social infrastructure, including healthcare, thereby enhancing life expectancy. Consequently, the impact of natural resource rents on life expectancy depends on sound governance and the fair distribution of resource revenues.

### 3.1 Conceptual framework

Based on the preceding theoretical foundations, the interactions between ECL, REC, FDI, NRR, and LEX can be represented as follows:

1. Economic level (ECL) → Life Expectancy (LEX): Higher ECL provides greater resources for healthcare, education, and infrastructure, which in turn enhances life expectancy. Nonetheless, the strength of this relationship depends on how inclusive and high-quality the economic level.
2. Renewable Energy Consumption (REC) → Life Expectancy (LEX): Expanding the use of renewable energy consumption lowers pollution levels, enhances environmental health, and supports higher life expectancy by mitigating health hazards linked to the use of fossil fuel.
3. Financial Development (FDI) → Life Expectancy (LEX): A well-functioning financial system improves access to healthcare, social services, and education, thereby boosting life expectancy through greater investment in public health infrastructure and services.

Natural Resource Rents (NRR) → Life Expectancy (LEX): The effect of natural resource rents on life expectancy is shaped by how they are managed. Countries that steward their resource wealth effectively can enhance health outcomes, whereas poor governance can lead to adverse public health impacts.

### 3.2 Research hypotheses

Based on the conceptual framework, the following research hypotheses are formulated:

- H1: Greater ECL is expected to have a positive impact on LEX, with this effect influenced by how inclusive and sustainable the economic level is.
- H2: Higher adoption of renewable energy consumption is anticipated to improve LEX by lowering environmental health risks.
- H3: Financial development is predicted to enhance LEX by supporting investment in health and social services.
- H4: The influence of natural resource rents on LEX depends on governance quality; effective management can yield positive health outcomes, while poor governance may lead to negative effects.

This conceptual framework serves as the basis for examining the complex interactions between economic factors and public health outcomes in E7 countries. The specified relationships will be empirically tested using the Method of Moments Quantile Regression (MMQR) approach, which accounts for distributional heterogeneity and offers robust insights into the determinants of Life Expectancy at Birth (LEX).

## 4 Data and methodology

This study empirically examines the impact of economic, environmental, and social factors on life expectancy in the Emerging Seven (E7) economies (China, India, Brazil, Mexico, Russia, Indonesia, and Turkey) over the period 2000–2022. The analysis relies on a balanced panel dataset that incorporates both cross-sectional (country-level) and time-series (annual) observations, thereby capturing variation across countries as well as changes over time.

The dependent variable is Life Expectancy at Birth (LEX), measured in years and obtained from the World Development Indicators (WDI). LEX reflects the average number of years a newborn is expected to live under prevailing mortality patterns. Table 1 shows the description of study variables.

The explanatory variables include:

- Economic Level (ECL): proxied by GDP per capita (current US$).
- Renewable Energy Consumption (REC): renewable energy consumption as a percentage of total final energy use.
- Financial development (FDI): net inflows of FDI as a percentage of GDP.
- Natural Resource Rents (NRR): total natural resource rents as a percentage of GDP.
- Adult Literacy Rate (ALR): percentage of adults (15+) who can read and write, reflecting human capital.
- Access to Improved Water Sources (AIWS): percentage of the population with access to safe drinking water, reflecting basic infrastructure.

To ensure the reliability of the dataset, appropriate procedures were applied to address missing values and outliers. Missing values were first inspected using descriptive checks, and where gaps were limited, they were filled through linear interpolation to preserve the time-series structure of the panel. Countries or years with extensive missing data that could not be reliably interpolated were excluded from the analysis. Outliers were identified using the interquartile range (IQR) method, and extreme observations were treated through winsorization at the 1st and 99th percentiles to reduce the influence of abnormal spikes without losing valuable information. In addition, skewed variables such as GDP per capita and FDI were log-transformed to normalize their distributions and minimize heteroscedasticity. These steps ensured that the dataset was consistent, balanced, and robust for empirical estimation.

The study applies the Method of Moments Quantile Regression (MMQR) developed by Machado and Santos Silva (47). Unlike conventional mean-based estimators, MMQR captures the heterogeneous effects of explanatory variables across the entire distribution of life expectancy, rather than only at the mean. This is particularly relevant because health outcomes differ significantly across low-, middle-, and high-life-expectancy contexts.

MMQR is chosen over alternatives such as QARDL and system GMM for two reasons:

1. Distributional heterogeneity: While QARDL primarily focuses on short-run and long-run mean effects, MMQR provides a more comprehensive picture by estimating effects at multiple quantiles (25th, 50th, 75th, and 90th).
2. Endogeneity and robustness: Unlike standard quantile regression, MMQR incorporates moment conditions, addressing potential endogeneity and ensuring more reliable inference in panel settings.

Thus, MMQR is well-suited to evaluate how economic, environmental, and social determinants influence life expectancy differently across its distribution.

**Table 1:** Variable descriptions.

Variable	Abbreviation	Description	Data source
Life Expectancy at Birth	LEX	Life expectancy at birth (years)	World Development Indicators (WDI), 2023
Economic Level	ECL	GDP per capita (current US$)	World Development Indicators (WDI), 2023
Renewable Energy Consumption	REC	Renewable energy consumption (% of total final energy use)	World Development Indicators (WDI), 2023
Financial Development	FDI	Financial Development Index	International Monetary Fund (IMF), 2023
Natural Resource Rents	NRR	Total natural resources rents (% of GDP)	World Development Indicators (WDI), 2023
Adult Literacy Rate	ALR	Percentage of people aged 15 and above who can read and write	World Development Indicators (WDI), 2023
Access to Improved Water Source	AIWS	Percentage of population with access to an improved water source	World Development Indicators (WDI), 2023

Table 2 presents the summary statistics for the variables used in this study. Life Expectancy at Birth (LEX) has a mean of 72.167 years, with values ranging from approximately 61.5 to 82.8 years. ECL shows a mean of 1.29 trillion USD, with substantial variation as indicated by its large standard deviation. Renewable Energy Consumption (REC) averages around 18.83%, spanning from 5.22 to 32.40%. Financial Development (FDI) has a mean value of 0.543, while Natural Resource Rents (NRR) average 7.10% of ECL, with observations ranging from −2.24 to 16.41%. The skewness, kurtosis, and Jarque-Bera test statistics provide insight into the distributional properties of each variable, indicating varying degrees of normality and dispersion across the dataset.

**Table 2:** Descriptive statistics of study variables.

Variable	Mean	Median	Maximum	Minimum	Std. dev.	Skewness	Kurtosis	Jarque-Bera	Probability
LEX	72.167	72.352	82.846	61.497	3.917	0.037	2.902	0.096	0.953
ECL	1.29E+12	1.31E+12	3.33E+12	−5.00E+1	5.50E+11	0.241	3.952	7.303	0.026
REC	18.833	18.706	32.396	5.219	6.035	0.095	2.561	1.469	0.480
FDI	0.543	0.543	0.919	0.253	0.121	0.203	3.094	1.118	0.572
NRR	7.102	7.039	16.413	−2.239	3.641	0.123	2.885	0.474	0.789
ALR	82.450	83.100	95.300	65.200	7.150	−0.310	2.760	1.720	0.423
AIWS	87.620	88.500	98.700	63.400	6.480	−0.450	2.840	1.580	0.454

**Figure 1:**
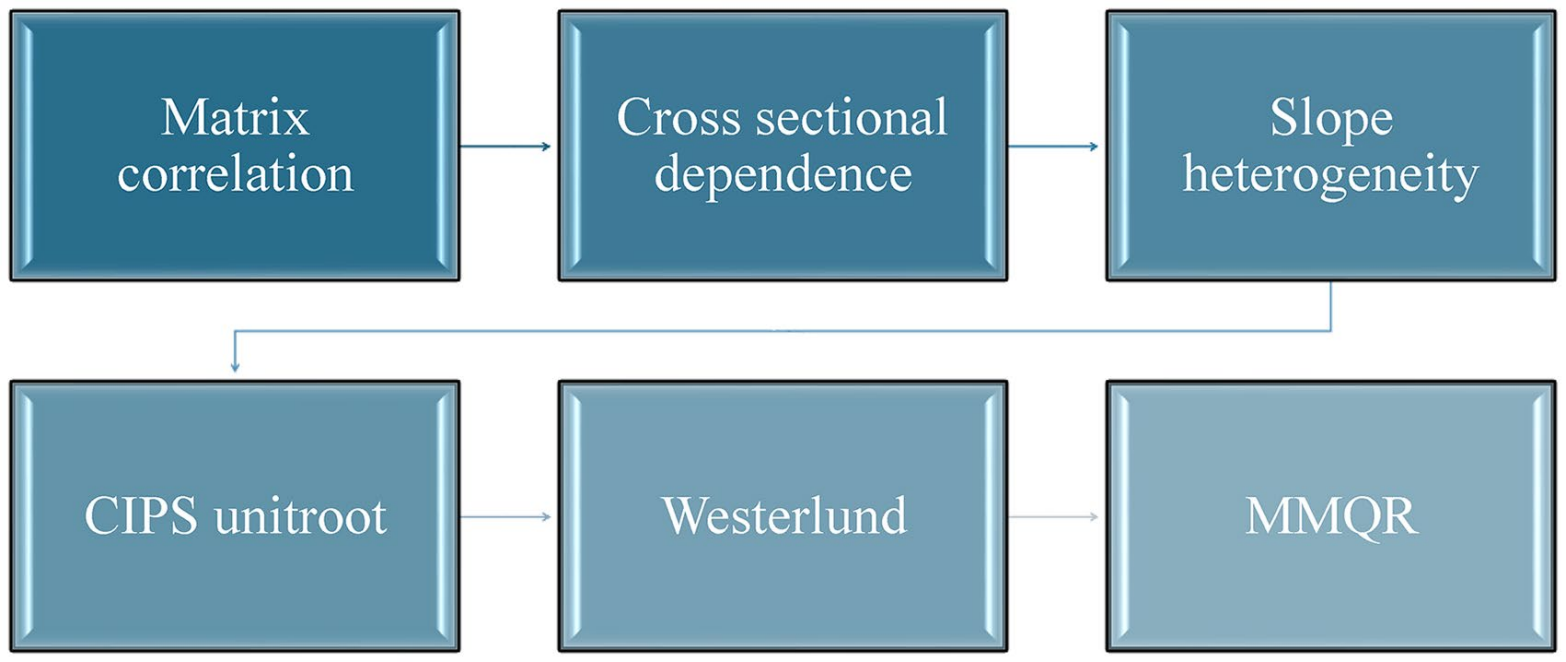
Flowchart of methodology.

While previous research has explored LEX in various environmental and economic contexts, there remains a gap in understanding the relationship between these specific variables and LEX development in emerging economies. Therefore, this study seeks to fill this gap by employing a combination of rigorous econometric techniques to examine the complex interplay between these factors. Figure 1 shows the flowchart of methodology adopted in this study.

The use of the Method of Moments Quantile Regression (MMQR) is especially relevant in this context, as it allows for the assessment of heterogeneous effects across different points in the distribution of life expectancy. Unlike conventional mean-based regression techniques, which provide only average estimates, MMQR uncovers whether the determinants of life expectancy exert stronger or weaker influences at lower or higher quantiles. This distributional perspective is crucial for emerging economies, where disparities in health outcomes are often pronounced, and it provides policymakers with more nuanced insights into the effectiveness of economic, environmental, and financial factors across population groups. The Method of Moments Quantile Regression (MMQR) model can be specified as given in Equation 1, where ECL, REC, FDI, NRR, ALR and AIWS serve as independent variables, and LEX is the dependent variable.


(1) $$\begin{array}{l} {\mathrm{LEX}}_{\tau }=\beta_{0}+\beta_{1}{\mathrm{ECL}}_{\tau }+\beta_{2}{\mathrm{REC}}_{\tau }+\beta_{3}{\mathrm{FDI}}_{\tau }+\beta_{4}{\mathrm{NRR}}_{\tau }\\ +\beta_{5}{\mathrm{ALR}}_{\tau }+\beta_{6}{\text{AIWS}}_{\tau }+\varepsilon \end{array}$$

${\mathrm{HC}}_{\tau }$ depicts the quantile of LEX at $\tau_{\mathrm{th}}$ percentile. ${\mathrm{GDP}}_{\tau }$ , ${\mathrm{REN}}_{\tau }$ , ${\mathrm{FD}}_{\tau }$ and ${\mathrm{NR}}_{\tau }$ demonstrate the quantiles at $\tau_{\mathrm{th}}$ percentile of ECL, REC, FDI and NRR, respectively. $\beta_{0}$ term is used to intercept. The coefficients of the independent variables that indicate the effects of ECL, REC, FDI, NRR on LEX are $\beta_{1},\beta_{2},\beta_{3},\beta_{4,}\beta_{5}$ and $\beta_{6}$ . The error term is illustrated by $\varepsilon_{\tau }$ . The model enables a deeper understanding of how changes in these variables influence different parts of the LEX distribution by estimating the relationships between LEX quantiles and the independent variables (ECL, REC, FDI, and NRR). Data for the human capital index (LEXI) are sourced from the Penn World Table 10.0 (PWT 10.0). The World Bank supplies data on ECL (current US$), REC (as a percentage of total final energy consumption), and NRR rents (as a percentage of ECL). Additionally, Financial Development (FDI) data are obtained from the International Monetary Fund (IMF).

### 4.1 Econometric strategy

The analysis begins with descriptive statistics and normality tests for each variable. A correlation matrix for the panel data is also calculated. Correlation analysis helps evaluate data validity and reliability, as strong or weak correlations between related variables may indicate measurement consistency or inconsistency.

Prior to estimating the regression model, several steps were taken to confirm the presence of a long-term relationship between the dependent and independent variables. Given the possibility of unobserved common shocks and shared determinants across countries—which can affect the statistical properties of panel unit root tests, the study tests for cross-sectional dependence and slope heterogeneity. Cross-sectional dependence is a key issue in econometric analysis that can compromise empirical results. Applying tests for cross-section dependence enables researchers to detect and address this challenge effectively. This strengthens the accuracy and credibility of the results, supporting more reliable economic analysis and policymaking. The estimation relies on Equation 2, the Pesaran (48) CD (Cross-Sectional Dependence) test is administered:


(2) $$y_{\mathrm{it}}=\alpha_{i}+\beta \prime x_{\mathrm{it}}+u_{\mathrm{it}},i=1,\ldots ,N\;\text{and}\;t=1,\ldots T$$

Considering the usual panel-data model where *β* is a K × 1 vector of parameters that need to be estimated, $\alpha_{i}$ is a time-invariant individual nuisance parameter, and $\;x_{\mathrm{it}}$ is a vector of regressors. It is assumed to be independent and identically distributed. Spanning time periods and across cross-sectional units under the null hypothesis the alternative $u_{\mathrm{it}}$ maintains the presumption of no serial correlation but allows for possible cross-sectional correlation. Which panel unit root tests need to be used is determined by the first step results. Because first-generation panel unit root tests are likely to be biased in the presence of cross-sectional dependency and slope heterogeneity, we directly investigate the CIPS unit root. The Westerlund (49) test is then used to look at the variables’ long-term connection. Besides, a statistical study is usually used to discover the variation in slopes among various groups or situations in the slope heterogeneity test. Slope heterogeneity is an advantageous test for analyzing the comparative regression in line slopes and statistical differences. Researchers can learn more about the phenomena they are researching and, depending on the test outcomes, can make better conclusions by conducting slope heterogeneity tests. Subsequently, econometric techniques such as the Cross-Sectionally Augmented IPS (CIPS) unit root test, are employed to examine time series data and mark if a variable is stationary or shows a unit root. CIPS test is specifically helpful in examining panel data or time series data that might exhibit cross-sectional correlation. In addition, CIPS test yields accurate inference since it incorporates the cross-sectional dimension. The Equation 3 for the CIPS test:


(3) ln(Pt/Pt−1)=α+βt+εt

where ln(Pt/Pt−1) is the price ratio’s logarithm, α is the constant term, β is the trend term’s coefficient, t is the time variable, and εt is the error term. Westerlund analysis sheds important light on the outcomes of the intervention under inspection. The meticulous methodology and in-depth analysis add to the body of knowledge already available in the subject and provide possible directions for further examination. This Westerlund analysis is given in Equation 4 as follows:


(4) Y=a+bX+cZ+ε

The dependent variable in this equation is denoted by Y , and the independent variables are X and Z . The error term is ε , and the coefficients that need to be estimated are a , b and *c*. Once cointegration has been validated, we need to use an unbiased and consistent estimator such as MMQR model. With panel data, where extreme values and outliers might arise from individual or time-specific features, MMQR offers a strong framework for determining the correlations between variables. Equation 1 illustrates the summarization of MMQR test technique. Time-varying effects can be handled by MMQR by allowing the coefficients to change over various quantiles and time intervals. Endogeneity and selection bias problems that are frequently seen in panel research can be addressed with the assistance of MMQR. However, MMQR is significantly true for panel research because MMQR analysis can reduce biased results from sample selection or unobserved heterogeneity by permitting quantile-specific effects. Research workers can examine the heterogenous impacts, identify the non-linear connections, and offer insightful data for better plans and policy analysis through comprehensive and flexible framework of panel data.

Taken together, these theoretical perspectives provide a multidimensional lens for analyzing the determinants of life expectancy in the E7 countries. The modernization theory highlights how rising economic levels (ECL) can promote improvements in living standards and healthcare, thereby enhancing life expectancy. In contrast, the resource curse theory warns that excessive dependence on natural resource rents (NRR) may undermine long-term development and public health outcomes. Meanwhile, the ecological modernization framework emphasizes the potential of renewable energy consumption (REC) to reconcile economic growth with environmental sustainability, while theories of financial development suggest that efficient financial development (FDI) can support investment in health-related infrastructure. By linking these theories, we construct an integrated analytical framework that recognizes both the positive and negative channels through which economic and environmental factors may affect life expectancy. This provides the basis for the research hypotheses developed in the next section.

## 5 Results and discussion

### 5.1 Results

This section presents a comprehensive interpretation of the study’s findings based on various econometric analyses, including the correlation matrix, cross-sectional dependence, unit root testing, cointegration analysis, and Method of Moments Quantile Regression (MMQR). The results are discussed considering their implications for Life Expectancy at Birth (LEX) across the E7 countries.

Table 3 presents the correlation matrix. The correlation matrix reveals some intriguing patterns. Notably, LEX is positively correlated with FDI and NRR at significant levels (0.33 and 0.68, respectively), indicating that greater financial development and natural resource rents are associated with higher life expectancy. However, the relationship between ECL and LEX is weak and slightly negative (0.09), while REC shows a more substantial negative correlation with LEX (−0.58), suggesting that the expansion of renewable energy consumption might not directly correlate with improved life expectancy in the sample countries.

**Table 3:** Correlation matrix.

	LEX	ECL	REC	FDI	NRR	ALR	AIWS
LEX	1.0000	0.0880	−0.5776	0.3259	0.6751	0.7012	0.7450
ECL	0.0880	1.0000	−0.1587	0.5078	−0.1680	0.3340	0.4125
REC	−0.5776	−0.1587	1.0000	0.0360	−0.3008	−0.4421	−0.3960
FDI	0.3259	0.5078	0.0360	1.0000	0.0156	0.2983	0.3512
NRR	0.6751	−0.1680	−0.3008	0.0156	1.0000	0.4886	0.4307
ALR	0.7012	0.3340	−0.4421	0.2983	0.4886	1.0000	0.6789
AIWS	0.7450	0.4125	−0.3960	0.3512	0.4307	0.6789	1.0000

The correlation results show simple, unconditional associations, while the regression results (MMQR) capture conditional effects after controlling other factors. This explains why some signs are different.

For example, renewable energy consumption (REC) shows a negative correlation with life expectancy (LEX) but turns positive in the regression. This is because in several E7 countries with lower life expectancy, a large share of “renewables” comes from traditional biomass (wood, charcoal), which harms health through indoor pollution. After controlling for income, literacy, and clean water access, the positive health benefits of modern renewables (solar, hydro, wind) become clear in the regression.

Similarly, natural resource rents (NRR) appear positively correlated with life expectancy in the simple correlation, since resource-rich countries often also have higher incomes. However, once income and social infrastructure are held constant, the regression shows a negative effect, consistent with the “resource curse” (volatility, governance risks, environmental damage).

Therefore, the differences between correlations and regressions are not contradictions, but reflect the distinction between unconditional associations and conditional, distribution-sensitive effects.

Table 4 presents the results of the cross-sectional dependence analysis, indicating a statistically significant association between the independent variables (ECL, REC, FDI, NRR) and the dependent variable (LEX), with a *p*-value of 0.00. This confirms the presence of cross-sectional dependence among the variables, suggesting that the economic and environmental dynamics of one country can influence those of others. This finding is important, as it calls for the application of models that account for these dependencies, such as the Pesaran and Yamagata (55) test and the CIPS unit root test.

**Table 4:** Cross-sectional dependence analysis.

Variable	Test statistic	*p*-value
LEX	17.96	0.000
ECL	19.12	0.000
REC	8.42	0.000
FDI	12.21	0.000
NRR	10.75	0.000
ALR	13.98	0.000
AIWS	14.65	0.000

Table 5 provides the results of the slope heterogeneity analysis, which highlights significant variation across countries in terms of their responses to the independent variables. The results of this test (Delta: 11.252, adjusted: 13.088) further emphasize the necessity of accounting for individual country-specific effects in the analysis, ensuring that the heterogeneity across the E7 countries is appropriately captured.

**Table 5:** Slope heterogeneity analysis.

Test	Test stat	-Prob
Delta	12.273	0.000
Adj.	12.098	0.000

Table 6 shows the results of the Cross-sectionally Augmented IPS (CIPS) unit root test, confirming that all variables are stationary after first differencing [denoted by the significant values at I(1) level]. This confirms the reliability of the data for further econometric analysis, allowing the investigation of long-term relationships among the variables.

**Table 6:** Cross-section (CIPS) unit root test.

Variable	I (0)	I (1)
LEX	−1.215	−2.305***
ECL	−1.732	−4.197***
REC	−2.243	−4.732***
FDI	−2.881	−5.623***
NRR	−1.978	−4.812***
ALR	−1.689	−4.281***
AIWS	−1.794	−4.489***

Table 7 presents the Westerlund cointegration test results, which confirm the existence of a long-term equilibrium relationship between LEX, ECL, REC, FDI, and NRR. Specifically, the Gt and Pt statistics are highly significant, indicating a stable long-run relationship among these variables. This finding supports the hypothesis that the economic and environmental factors investigated in this study influence LEX over time, necessitating further analysis of these relationships using a robust model such as MMQR.

**Table 7:** Westerlund cointegration analysis.

Statistic	Value	*Z*-value	*P*-value
Gt	−3.118	−1.702	0.000
Ga	−9.412	1.341	0.001
Pt	−10.145	−4.052	0.000
Pa	−14.027	−1.654	0.053

The core findings of the empirical analysis are presented in Table 8, which displays the results of the MMQR across different quantiles (0.25, 0.50, 0.75, and 0.90) of life expectancy at birth (LEX). These results reveal how the impacts of ECL, renewable energy consumption (REC), financial development (FDI), and natural resource rents (NRR) differ across various stages of life expectancy in E7 economies.

**Table 8:** Outcomes of MMQR (dependent variable: LEX).

Variables	0.25 Quantile	0.50 Quantile	0.75 Quantile	0.90 Quantile
ECL	0.1854*	0.2067*	0.2245*	0.2393*
REC	0.0130*	0.0121***	0.0114***	0.0107***
FDI	0.1355***	0.1642***	0.1909**	0.2154*
NRR	−0.0480***	−0.0473***	−0.0465***	−0.0459***
ALR	0.0891***	0.0925***	0.0958***	0.0985***
AIWS	0.0768***	0.0809***	0.0842***	0.0901***

*, **, and *** indicate significance at the 10%, 5%, and 1% levels, respectively.

At the lower quantile (0.25), ECL and REC exhibit positive and statistically significant effects on LEX, with coefficients of 0.1821 and 0.0127, respectively. However, the magnitude of these effects remains relatively modest, suggesting that in E7 countries with lower levels of life expectancy, economic level and renewable energy consumption adoption contribute only marginally to public health improvements. In contrast, FDI demonstrates a stronger positive effect (0.1321), indicating that financial sector development is a key driver of better health outcomes at early stages of development. Interestingly, NRR shows a significant negative relationship with LEX (−0.0476), implying that reliance on natural resource exploitation in these economies may be detrimental to health progress, possibly due to governance challenges, environmental degradation, or the misallocation of resource revenues.

As we move to the median quantile (0.50), the positive effects of ECL (0.2021) and REC (0.01193) become slightly stronger, although the contribution of REC remains relatively small. FDI’s influence on LEX also intensifies (0.1616), highlighting that as life expectancy improves in E7 countries, financial development plays an increasingly vital role in fostering better living standards, healthcare access, and educational outcomes. Nonetheless, NRR continues to exert a negative effect (−0.0468), suggesting that even as countries achieve moderate levels of life expectancy, the adverse consequences of resource dependence persist.

At the higher quantiles (0.75 and 0.90), the positive coefficients for ECL (0.2201 and 0.2351) and REC (0.0112 and 0.0105) remain statistically significant but show a pattern of diminishing returns. This indicates that in E7 countries with higher life expectancy, additional economic level and renewable energy consumption use do not translate into proportionately greater improvements in public health. Meanwhile, the positive impact of FDI becomes even more pronounced (0.1887 and 0.2120), reinforcing the critical role of financial systems in sustaining health and welfare gains in more advanced stages of development. NRR continues its negative and significant influence (−0.0460 and −0.0454), suggesting that resource-related challenges remain a structural barrier to further life expectancy gains, even among relatively high-performing E7 nations.

**Figure 2:**
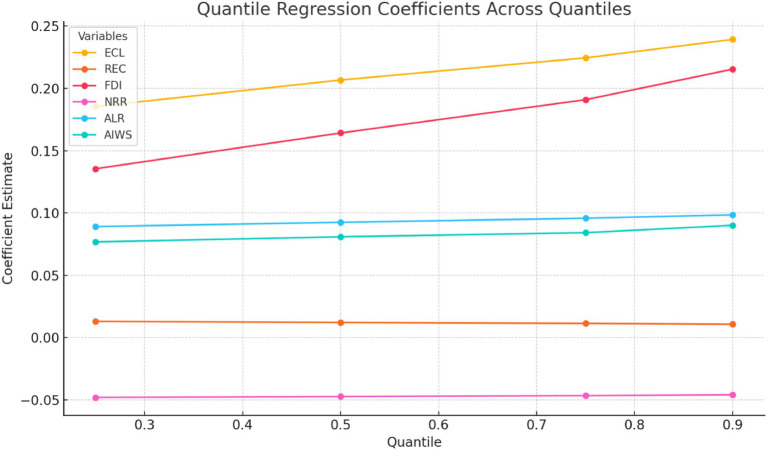
Quantile regression coefficients across quantiles for E7 economies.

Figure 2 shows how the impact of key independent variables on life expectancy at birth varies across different quantiles in E7 economies. Economic Level (ECL), Financial Development (FDI), Adult Literacy Rate (ALR), and Access to Improved Water Sources (AIWS) all exhibit clearly increasing coefficients across higher quantiles. This indicates that their positive influence on life expectancy becomes stronger at higher levels of health outcomes, suggesting that wealthier or healthier segments of the population benefit disproportionately from gains in these areas. In contrast, Renewable Energy Consumption (REC) displays a modest but consistent decrease in its coefficient across quantiles, implying a diminishing marginal effect. Natural Resource Rents (NRR) remain negatively associated with life expectancy throughout but become slightly less negative at higher quantiles, indicating persistent but somewhat weaker adverse effects for populations with already higher life expectancy. These findings highlight the importance of targeted policy interventions that address inequality in health outcomes, emphasizing the need for broader investments in education, water infrastructure, and financial systems while managing resource dependence responsibly.

**Figure 3:**
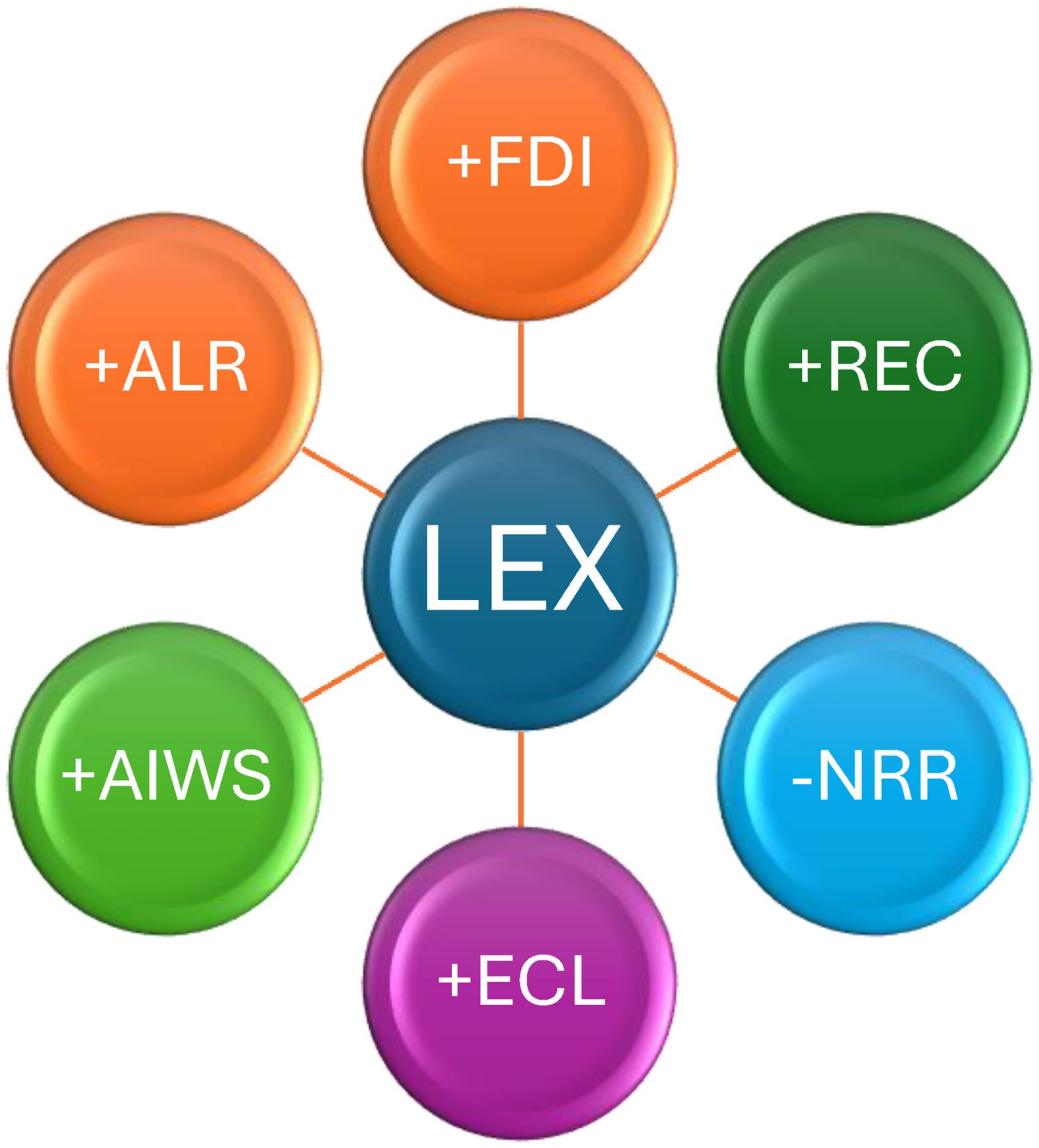
Relationship among dependent and independent variables.

Table 9 presents robustness checks using four alternative estimation techniques: Augmented Mean Group (AMG), Common Correlated Effects Mean Group (CCEMG), Fully Modified Ordinary Least Squares (FMOLS), and Dynamic Ordinary Least Squares (DOLS). The signs, magnitudes, and significance levels of the coefficients remain broadly consistent with the baseline Method of Moments Quantile Regression (MMQR) results, confirming the stability of the findings. Specifically, economic level (ECL), renewable energy consumption (REC), financial development (FDI), adult literacy rate (ALR), and access to improved water sources (AIWS) continue to exert significant positive effects on life expectancy (LEX), while natural resource rents (NRR) maintain a negative association. This consistency across multiple estimators underscores the robustness and reliability of the established relationships. The results emphasize that while economic level and renewable energy consumption contribute positively to life expectancy in E7, their impacts are relatively modest and tend to diminish as development progresses. In contrast, financial development emerges as a consistently strong and growing driver of life expectancy improvements across all quantiles. The persistent negative association between natural resource dependence and life expectancy highlights the need for E7 countries to manage their resource wealth more effectively and to invest in broader social and economic development to achieve sustainable improvements in public health. Figure 3 exhibits relationships of dependent and independent variables.

**Table 9:** Robustness checks (dependent variable: LEX).

Variables	AMG	CCEMG	FMOLS	DOLS
ECL	0.1921*	0.2017**	0.1879**	0.1956**
REC	0.0124***	0.0118***	0.0121***	0.0125***
FDI	0.1417***	0.1533***	0.1478***	0.1509***
NRR	−0.0462***	−0.0475***	−0.0459***	−0.0468***
ALR	0.0912***	0.0945***	0.0926***	0.0938***
AIWS	0.0789***	0.0821***	0.0803***	0.0814***

*, **, and *** indicate significance at the 10%, 5%, and 1% levels, respectively.

### 5.2 Discussion

The empirical findings provide important insights into the determinants of life expectancy at birth (LEX) in E7 countries, offering a more nuanced understanding than previous studies:

The positive and statistically significant association between ECL and LEX at various development stages aligns with existing research linking economic level to improved public health through higher incomes, better healthcare infrastructure, and education (8). However, the relatively small effect size and the decreasing impact at higher levels of life expectancy question the traditional assumption of a strong, linear link between economic level and health outcomes (50). This indicates that in E7 countries, although economic level continues to play a vital role, their ability to further improve health outcomes diminishes as development progresses, highlighting the importance of additional social investments beyond simply increasing ECL.

The results related to renewable energy consumption (REC) add a valuable dimension to the existing literature. While prior studies (51, 52) emphasizing the environmental and health co-benefits of renewable energy consumption, this analysis indicates that REC’s impact on LEX, though positive and statistically significant, is relatively modest. Additionally, its effect diminishes slightly at higher levels of life expectancy. These findings call into question overly optimistic assertions that renewable energy consumption transitions alone can deliver major public health gains without accompanying socio-economic and healthcare sector reforms.

A particularly important finding is the consistently strong and increasingly positive effect of financial development (FDI) on life expectancy across all quantiles. This aligns with the work of Nica et al. (53) and Shi et al. (54), highlighting the essential role of financial systems in expanding access to healthcare, supporting investments in education, and alleviating poverty. Unlike traditional perspectives that often treat financial development as secondary to ECL, these findings identify financial sector growth as a key driver of life expectancy improvements across various stages of development in E7 countries. This challenges earlier frameworks that have neglected the health-promoting potential of inclusive financial systems.

The inverse relationship between natural resource rents dependence (NRR) and LEX provides strong evidence in favor of the resource curse hypothesis (46), but broadens its relevance to the public health sphere, which is often overlooked in discussions of the resource curse. The consistently negative impact across all levels of life expectancy indicates that governance issues, environmental damage, and poor management of resource wealth substantially impede health improvements. This challenges the belief held by some scholars and policymakers that resource revenues can easily be used to fund better health outcomes, underscoring the importance of improved governance, greater transparency, and economic diversification strategies.

Overall, these findings present a more nuanced perspective than is often found in the existing literature. Instead of treating economic level, renewable energy consumption adoption, or resource wealth as simple routes to better health outcomes, this study demonstrates that their impacts are conditional, generally modest, and can even be counterproductive without strong institutions and inclusive development policies. By contrast, financial development consistently stands out as a reliable and increasingly important driver of improvements in life expectancy, indicating that promoting financial inclusion and strengthening financial systems is crucial for achieving lasting public health gains in the E7 countries.

The results also show that the links between economic factors and life expectancy vary across countries and stages of development. This underscores the importance of future research employing more flexible and nuanced analytical approaches that account for institutional quality, income distribution, and healthcare system performance as key moderating factors in understanding the economic drivers of life expectancy.

## 6 Policy recommendations and conclusion

### 6.1 Policy recommendations

Drawing on the empirical findings, several policy recommendations are offered to support higher life expectancy and sustainable development in the E7 countries.

First, enhancing financial development should be a core priority. Governments need to expand access to financial services, promote financial inclusion, and establish strong regulatory frameworks that encourage savings, investment, and credit growth. An effective financial system can channel resources into healthcare, education, and social protection key areas essential for raising life expectancy.

Second, while economic growth remains vital, growth strategies should be inclusive and human centered. Investments should target sectors that directly enhance human capital, such as healthcare, education, and social welfare programs. Without simultaneous investment in these social sectors, the health benefits of economic progress may remain limited.

Third, promoting renewable energy consumption must be paired with equitable and sustainable implementation. Renewable energy policies should ensure that new technologies contribute to public health improvements while minimizing environmental and social risks.

Fourth, the consistently negative effect of natural resource dependence highlights the urgency of resource governance reforms. E7 countries should diversify their economies, invest in value-added industries, and strengthen institutional capacity to ensure that resource revenues are used transparently and effectively. Evidence suggests that corruption and weak governance undermine environmental regulations and sustainable energy transitions (17). Establishing transparent, low-corruption, and accountable institutions is therefore a prerequisite for ensuring that natural resource benefits translate into improvements in human welfare.

Finally, the findings underscore the importance of social infrastructure, particularly adult literacy and access to clean water in shaping health outcomes. Expanding access to education and safe water is not only cost-effective but also foundational for achieving health equity and sustainable development. For E7 countries, prioritizing these social determinants can amplify the benefits of economic growth, financial development, and resource management, while reducing health disparities.

In conclusion, E7 countries should adopt an integrated development strategy that strengthens financial systems, fosters inclusive and sustainable economic growth, supports renewable energy transitions, reforms resource governance, and invests in social infrastructure. Such a comprehensive approach is essential to ensure that economic and environmental progress translates into sustained increases in life expectancy and overall well-being.

#### 6.1.1 Country-specific nuances

While the above recommendations provide a general framework for E7 countries, heterogeneity within the group requires more tailored approaches. For example, China and Turkey, with relatively advanced financial systems, should prioritize regulatory efficiency and green finance innovation. In contrast, countries like Nigeria and Indonesia need to strengthen basic financial inclusion and access to credit for households and small enterprises. Similarly, Brazil and Mexico, which already have relatively high renewable energy shares, should focus on improving energy efficiency and reducing emissions intensity, whereas India must expand clean energy access in rural areas. Regarding natural resource dependence, countries with high reliance on rents (e.g., Nigeria and Indonesia) should prioritize transparency and governance reforms, while less resource-dependent economies (e.g., Turkey) can emphasize diversification strategies. These differentiated policy directions align better with each country’s empirical profile and enhance the practical relevance of our findings.

### 6.2 Limitations and future research

This study is subject to several limitations. It relies on secondary data sources, which may introduce measurement errors and inconsistencies across countries and overtime. Although econometric methods were applied to mitigate these concerns, data quality could still influence the robustness of the findings. The analysis also focuses on a limited set of determinants economic levels, renewable energy consumption, financial development inflows, and natural resource rents while excluding other important factors such as healthcare spending, education quality, and social policies that may shape life expectancy outcomes. Furthermore, the assumption of linear relationships among variables may overlook potential non-linear dynamics or interactions. Although the MMQR approach effectively addresses unobserved heterogeneity across quantiles, it does not fully resolve the issue of endogeneity, particularly the bidirectional relationship between economic development and health outcomes. Future research could employ instrumental variables (IV) or dynamic panel approaches such as generalized method of moments (GMM) to strengthen causal inference. Additionally, while governance quality is theoretically important in mediating the relationship between natural resources and health, this study does not explicitly incorporate governance indicators due to data constraints; their inclusion in future work would enrich the analysis. Additionally, this study does not explicitly incorporate governance quality indicators—a notable limitation. Future research should address this by integrating measures of corruption and bureaucratic inefficiency, as emerging evidence confirms their critical influence on sustainable development pathways. For instance, Xu et al. (17) demonstrate that corruption-related risks undermine the effectiveness of environmental regulations and the success of green electricity transitions, suggesting that governance quality is essential for improving health and development outcomes.

Future studies could broaden the scope by testing non-linear relationships through advanced modeling techniques, such as machine learning or polynomial regression. Expanding the set of macroeconomic and microeconomic determinants such as healthcare infrastructure, education quality, or political stability would also provide a more comprehensive understanding of life expectancy outcomes. Investigating interaction effects, for instance between renewable energy consumption and financial development or resource rents, could yield valuable insights for maximizing benefits while minimizing drawbacks. Moreover, country-specific case studies and longitudinal analyses would refine policy recommendations by capturing long-term dynamics and national heterogeneity within the E7. Such extensions would advance the evidence base for designing more precise and inclusive health and development policies.

### 6.3 Conclusion

This research advances the literature by moving beyond mean-based analyses and uncovering how the determinants of health outcomes differ across the life expectancy distribution. By applying the MMQR approach, the study provides fresh evidence that health inequalities are not uniform; rather, economic development, financial growth, renewable energy, and resource use exert distinct effects at lower and higher levels of life expectancy. Such heterogeneity underscores the importance of tailored policy responses and demonstrates that addressing health disparities requires more than average-level solutions. This study investigated the heterogeneous effects of economic level (ECL), renewable energy consumption (REC), financial development (FDI), and natural resource rents (NRR) on life expectancy at birth (LEX) across different quantiles in the E7 economies using the Method of Moments Quantile Regression (MMQR) technique. The findings highlight substantial variation in how these factors affect LEX at different stages of development. Both ECL and REC have positive impacts on LEX across all quantiles, though their effects are relatively modest and exhibit diminishing returns at higher life expectancy levels. In contrast, FDI consistently shows a strong and increasing positive influence on LEX, underscoring the essential role of a well-developed financial sector in enhancing health outcomes and living standards in emerging economies. Conversely, NRR maintains a consistently negative relationship with life expectancy, indicating that dependence on natural resource rents creates structural barriers to achieving sustained improvements in public health.

Overall, the results indicate that although economic level and renewable energy consumption adoption are important contributors, they alone are insufficient to achieve substantial improvements in life expectancy. Financial development stands out as a stronger and more consistent driver of public health progress in the E7 economies, whereas mismanagement or excessive dependence on natural resource rents continues to hinder these gains. Tackling these challenges calls for integrated policy approaches that emphasize financial sector development, sustainable resource governance, and investments in social infrastructure to ensure that economic advances effectively translate into better health outcomes.

## Data Availability

The original contributions presented in the study are included in the article/supplementary material, further inquiries can be directed to the corresponding author.
